# Scale Deposition Inhibiting Composites by HDPE/Silicified Acrylate Polymer/Nano-Silica for Landfill Leachate Piping

**DOI:** 10.3390/ma13163497

**Published:** 2020-08-07

**Authors:** Min Li, Rui Zhao, Sude Ma, Tianxue Yang

**Affiliations:** 1 Faculty of Geosciences and Environmental Engineering, Southwest Jiaotong University, Chengdu 611756, China; lmscu@126.com; 2 School of Materials Science and Engineering, Xihua University, Chengdu 610039, China; masude2007@163.com; 3 State Key Laboratory of Environmental Criteria and Risk Assessment, Chinese Research Academy of Environmental Sciences, Beijing 100012, China; yangtianxue17@163.com

**Keywords:** Anti-scaling, surface free energy, Leachate, Pipe, silicified acrylate copolymer, HDPE

## Abstract

Scaling commonly occurs at pipe wall during landfill leachate collection and transportation, which may give rise to pipe rupture, thus posing harm to public health and environment. To prevent scaling, this study prepared a low surface energy nanocomposite by incorporating silicone-acrylate polymer and hydrophobically modified nano-SiO_2_ into the high-density polyethylene (HDPE) substrate. Through the characterization of contact angle, scanning electron microscopy and thermogravimetry, the results showed that the prepared composite has low wettability and surface free energy, excellent thermal stability and acid-base resistance. In addition, the prepared composite was compared with the commercial HDPE pipe material regarding their performance on anti-scaling by using an immersion test that places their samples into a simulated landfill leachate. It was apparent that the prepared composite shows better scaling resistance. The study further expects to provide insight into pipe materials design and manufacture, thus to improve landfill leachate collection and transportation.

## 1. Introduction

Landfill leachate has high chemical oxygen demand and complex constitutions, due to the large number of organic and inorganic pollutants contained [1]. Generally, it is transferred for advanced treatment by using a pipe system [2]. High density polyethylene (HDPE) is a common drainage material, which has been widely used for leachate piping, due to its excellently mechanical properties [3]. However, scaling often occurs at its inner wall during the piping, which may result in pipe rupture, thus posing a risk to human health and the environment [4].

The pipe scale formation is considered as a joint reaction between the leachate and piping materials [5]. To mitigate clogging consequences, conventional engineering measures have mainly focused on pipe cleaning, including aeration in the pipeline [6], adjustment of hydraulic loading [7], high-pressure water jetting [8], addition of scale inhibitors [9], etc. However, such engineering measures are constrained by their implementation of frequency and associated operation costs, which may not substantially prevent pipe scaling [9,10].

Development of anti-fouling materials has aroused great attentions in recent years regarding the reinforcement of long-term performance on sewage piping stability. A number of studies have indicated that minimization of material surface energy is effective at reducing pipe scale by using functional coatings [11,12,13]. Siloxane polymer and acrylate polymer are typical materials with low surface energy, which have been widely selected as substrates for coatings preparation due to their high bond energy, water repellency, and oxidation stability [14,15,16]. For instance, Yee et al. prepared polydimethylsiloxane coatings that demonstrated weak adhesion to the calcium carbonate deposition [17]. Ba et al. reported a polydimethylsiloxane fouling release coating, which is efficient at decreasing the adhesion of biofilm in sewage pipes [18]. Xie et al. grafted a telomer to prepare a bis-silanol terminated poly (dimethylsiloxane) as a silicone coating in the application of pipe antifouling [19]. Friis et al. applied methoxysilane compound as the precursor to improving the anti-scaling property of poly (oligo ethyleneglycol) methacrylate based coatings via surface-initiated polymerization [20].

Superhydrophobic nano silica, as a functional filler, is commonly used in preparation of the self-cleaning composite coatings because of its high stability and excellent compatibility with other basis materials [21,22]. Song et al. prepared heptadecafluorodecyltriisopropoxysilane (FPS)-SiO_2_ coatings on stainless steel substrates to improve anti-corrosion performance on geothermal water pipeline [23]. Wang et al. introduced super-amphiphobic coatings based on highly fluorinated and hierarchical-structure halloysite nanotubes (HNTs)/SiO_2_ powder that could be applied as self-cleaning substrates [24]. Qian et al. further added carbon nanofillers into fluorinated ethylene propylene and SiO_2_ composites to improve their scale-inhibition properties by using a test of saturated CaCO_3_ deposition [25].

However, the coatings may be easily peeled off due to different stress forces between substrates and coatings [12,26]. In particular, they may be not suitable for leachate pipes, due to the complex fluid-structure interaction [3]. This study aims to develop a low surface free energy acrylate copolymer to improve the anti-scaling performance on pipe material. Although HDPE pipes are prone to scaling during landfill leachate collection and transport, they are a commonly selected polymer matrix which can be integrated with functional fillers (e.g., inorganic fillers, nanoparticles, coupling agents, etc.) to improve their performances on specific engineering practice [27,28,29]. The copolymer and hydrophobically modified nano-SiO_2_ in this study are used as HDPE fillers to prepare nanocomposites with different components. The performances on morphology and thermal behavior of the composites are characterized by scanning electron microscopy (SEM) and thermogravimetric analysis (TGA). The surface free energy test and the leaching experiment by placing the prepared composite into a simulated landfill leachate are implemented to investigate the anti-scaling performance. It is expected that this study may provide insight into the scaling prevention during the transport in the pipe, to ensure safe operation of landfill disposal.

## 2. Materials and Methods

### 2.1. Materials

Butyl methacrylate (BMA), glycidyl methacrylate (GMA), azodiisobutyronitrile (AIBN) and other inorganic reagents were purchased from Kelong Corporation Ltd., Chengdu, China. Vinyltrimethoxysilane (VTMO) and nanosilica were purchased from Keyan Corporation Ltd. Shanghai. High density polyethylene (HDPE) resin was obtained from SINOPEC Corporation Ltd. HDPE pipe materials were purchased from a local Pipe Supplier in Chengdu City, China with an internal diameter of 48 mm and a wall thickness of 2.0 mm. The initiator, Azodiisobutyronitrile (AIBN), was purified by recrystallization using absolute ethanol.

### 2.2. Experiment Design

**Figure 1:**
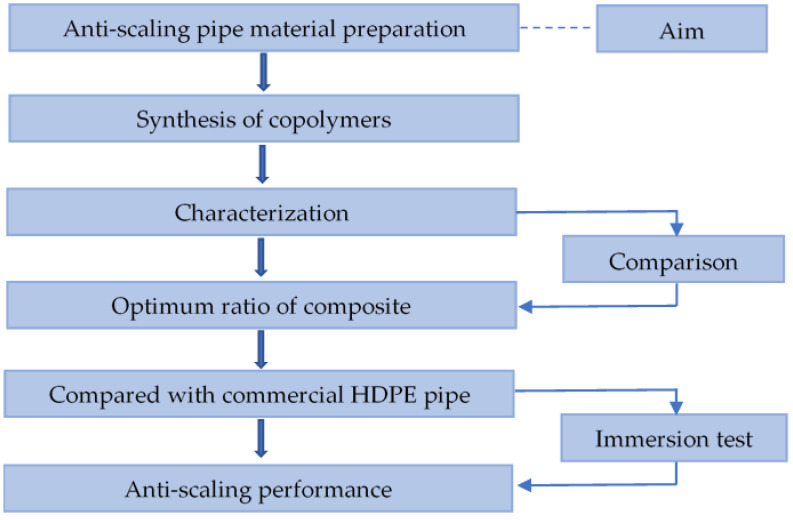
Experiment design for the composite preparation and characterization.

The experiment design is shown in Figure 1. First, the low surface energy copolymers were synthesized by selection of appropriate polymerization monomers. Then the Fourier transform infrared (FTIR) test, contact angle (CA) measurement, thermal analysis and SEM were used to characterize the prepared copolymers in order to identify the optimal composition for the synthesis. Through an immersion experiment where the prepared composite was placed into the simulated landfill leachate, the composite performance on scaling resistance was compared with that of the commercial HDPE pipe.

### 2.3. Preparation of Silicified Acrylate Copolymers

The polymers of silicified acrylate copolymer (abbreviated to PSAs) were synthesized by bulk free radical polymerization, and the preparation details were given as follows. A quarter of the mixture of monomer VTMO and BMA was added to a neck flask with agitator, reflux condenser, thermometer and drip funnel, and the initiator AIBN with 0.3% of the mass of the monomer was then added. The solution was degassed with nitrogen, and the reaction mixture was stirred at 75 °C for 30 min. The rest of the mixture of VTMO and BMA, GMA and the initiator AIBN with 0.2% of the mass of the monomer were added into the flask one by one. After dropping, the reaction mixture was kept at 75 °C for 40 min. Once the temperature of the reaction system began to rise, the heating was stopped. The mixture was transferred to a plate and polymerized at 70 °C for 20 h, then heated to 120 °C for 2 h. Table 1 shows the chemical compositions of different mixtures predefined in this study.

### 2.4. Preparation of Superhydrophobic VTMO-SiO_2_

A quantity of 2.5 g of SiO_2_ was dispersed into 100 mL of pure ethanol, and stirred for 30 min, to obtain a SiO_2_ suspension with a mass concentration of 2.5%. A quantity of 10 g VTMO was dissolved into 80 mL of ethanol solution (EtOH/H_2_O = 3:1), and acetic acid was used to adjust its pH to 4. They were stirred for 30 min, to prepare the hydrating solution for VTMO. Then, the prepared suspension of VTMO-SiO_2_ was further mixed with the VTMO solution, by which ammonia solution was used to adjust its pH to 10. This reaction was implemented by the magnetic stirring at 60 °C for 3 h. Once the reaction had been completed, the mixture was centrifuged by the speed of 10,000 r/min and lasted for 10 min, then repeated washing with pure ethanol and centrifuged for 3 times, to obtain a modified wet sample of VTMO-SiO_2_. The wet sample was dried at 80 °C for 2 h to prepare the VTMO-SiO_2_ powder.

### 2.5. Preparation of Composite Material

**Figure 2:**
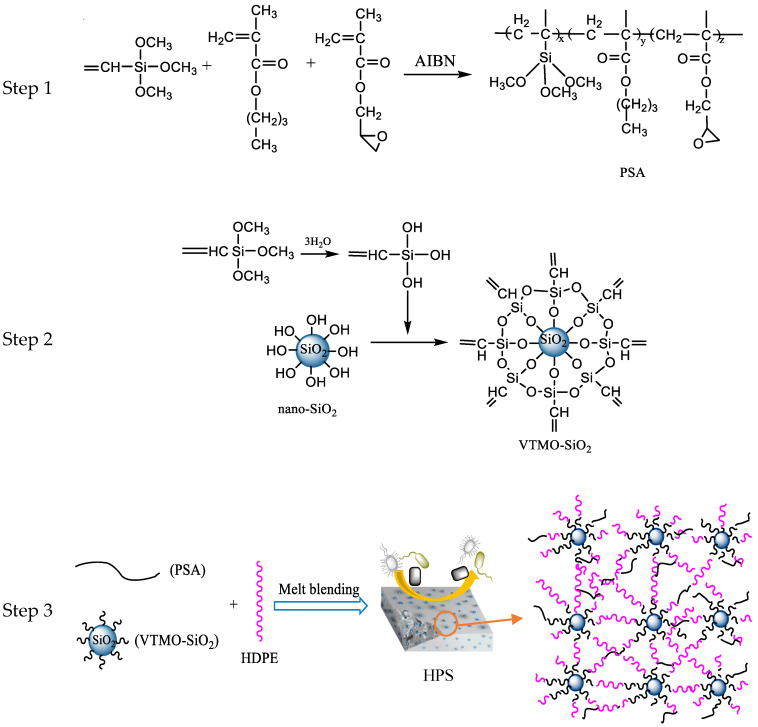
The preparation procedure for the HDPE/PSA/SiO_2_ composite.

Table 2 shows the different mass ratios of PSA, VTMO-SiO_2_ and HDPE pellets for the composite preparation. The composite containing 1% VTMO-SiO_2_ was entitled HDPE/PSA/SiO_2_(1%) (HPS1), and similar nomenclature was used for other proportions of the VTMO-SiO_2_. The premixed materials were compounded by the rotating and meshing twin-screw extruder, by which the temperature was set at 190 °C. The preparation process was shown in Figure 2.

### 2.6. Characterization

FTIR spectra is investigated by a Bruker Vector22 FTIR spectrometer within 400 cm^−1^ and 4000 cm^−1^. KBr powder was mixed with the samples, which were pressed into the flakes.

The static contact angle (CA) is tested by the DSA25 (KRÜSS GmbH, Hamburg, Germany) typed contact angle tester. The static contact angles in five different positions were investigated, and the average result was reported. The droplet volume was 4 μL used in the contact angle tester. Surface free energy is derived from the measurement of contact angles, given as follows [30]:

(1) $$\sigma_{solid}=\sigma_{solid}^{d}+\sigma_{solid}^{p}$$

(2) $$\sigma_{liquid}(1+\cos\theta )=2\sqrt{\sigma_{solid}^{d}\sigma_{liquid}^{d}}+2\sqrt{\sigma_{solid}^{p}\sigma_{liquid}^{p}}$$

where σ is the surface free energy, $\sigma^{d}$ and $\sigma^{p}$ denote the surface energy contributions from the dispersive and the polar component; $\sigma_{solid}^{d}$ and $\sigma_{solid}^{p}$ are measured by the contact angles (*θ*) between the solid surface and two probing liquids (H_2_O and CH_2_I_2_) of the known surface energy components ($\sigma_{liquid}^{d}$, $\sigma_{liquid}^{p}$).

The surface microstructure is investigated by field emission scanning electron microscope (SEM) (QUANTA FEG250, FEI). A digital camera by Huawei Corporation Ltd. (Shenzhen, China) was used to capture optical images of the sample. Thermogravimetric analysis (TGA) and derivative thermogravimetry (DTG) are performed by using the thermal gravimetric analyzer (TG209F1, NETZSCH Group, Bavaria, Germany) in a temperature range between 30 °C and 600 °C (10 °C/min) under a dynamic N_2_ atmosphere.

To study the anti-scaling performance on the prepared composite, an immersion experiment is designed. Although landfill leachate may have different characteristics, they are generally highlighted by high chemical oxygen demand, high salinity and high colority [1]. Moreover, existing studies indicated that the main scaling composition in the leachate pipe was calcium carbonate [4,31]. In such a context, the design of main parameters related to the water quality of the simulated leachate is given in Table 3. The leachate was made by reagents of analytical grade and deionized water, in which the addition of the reagents was determined by the simulated leachate concentration. Among them, chemical oxygen demand (COD) was prepared by glucose (1 g glucose equivalent to 1.067 g COD), calcium ion (Ca^2+^) by CaCl_2_, and suspended solids by humus. The pH of the leachate in 4.0 and 9.18 was adjusted by adding phosphate and borax buffer, respectively. The composite and commercial HDPE tubes were cut into samples respectively, with the same weight and the same inner wall area size, placed into the same prepared simulated leachate, to investigate their scaling performances with different immersing times.

## 3. Results and Discussion

### 3.1. Contact Angle and Surface Energy

The contact angles of different silicified acrylate polymers (PSAs) are presented in Table 4. The CA of the polymer is 92.1° without adding VTMO monomer. As the silicon monomer content increases, the CA of the polymer raises gradually. When the content of VTMO monomer increases to 10%, the CA reaches the peak of 104.9°. When the addition of VTMO continues increasing, the CA decreases slightly, and its change is not significant. The prepared polymer shows the optimal surface hydrophobic property with 10% VTMO content. Therefore, PSA10 is used for the preparation of composite.

The CA and surface free energy regarding different materials are presented in Table 5. It is clear that the surface free energy of pure HDPE is 37.0 mN/m. When the addition of hydrophobic SiO_2_ is increased from 1% to 5%, the wettability and surface free energy of the prepared composite are gradually deceased from 37.0 mN/m to 23.5 mN/m. With continuing to increase addition of SiO_2_, the wettability and surface free energy of the composite have not varied. This indicates that the addition of SiO_2_ at 5% is the optimal addition for the composite.

**Figure 3:**
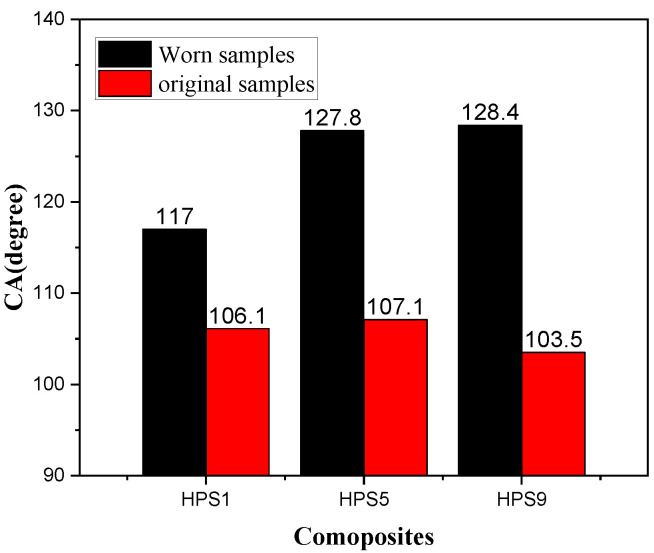
Change in contact angle (CA) as various nano-silica loadings for the HPS composites.

As the suspended solids in wastewater may cause wearing on pipe wall, it is necessary to investigate the wear resistance of the prepared material. The CA of the HPS composites with adding different mass ratio of VTMO-SiO_2_ is shown in Figure 3. It is identified that the CA of the composite is increased by 600 mesh sandpaper, and its hydrophobic property thus improves. When the hydrophobic silica content is 5%, the CA is increased to 127.8°. This may be due to the fact that more nano columnar structures are formed on the surface of the sanded composites. The valleys and grooves in the micro-nano surface may give rise to air cushion to expand the area for air-liquid contact, thus increasing the contact angle of the solid surface [33].

### 3.2. Fourier Transform Infrared (FTIR)

**Figure 4:**
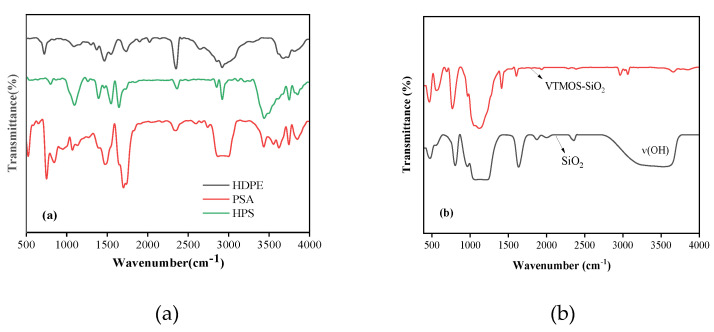
Fourier transform infrared (FTIR) spectra of different materials (**a**) pure high-density polyethylene (HDPE), copolymer PSA and composite HPS; (**b**) nano silicon.

The FTIR spectrum of the silicone-acrylate polymer and composites are shown in Figure 4. According to the PSA curve (Figure 4a), the bands at 2856 cm^−1^ and 2995 cm^−1^ are identified as C-H stretching vibrations of -CH_2_ and -CH_3_, respectively [34]. The carbonyl C=O stretching vibration occurs at an absorption peak of 1738 cm^−1^, whilst the epoxy group symmetrical stretch vibration occurs at an absorption peak of 854 cm^−1^. The bands at 1068 cm^−1^ and 1139 cm^−1^ are assigned to the Si-O symmetric stretching vibration and Si-O asymmetric absorption, respectively [35]. No obvious absorption peak is observed at 1640 cm^−1^ in the curve, indicating that the C=C double bond has disappeared and the monomer has polymerized. Compared with the HDPE sample, the prepared composite displays an apparent Si-O-C with absorption peaks at 790 cm^−1^, and the strong and wide absorption band is Si-O-Si antisymmetric stretching vibration at 1109 cm^−1^ [36], which indicates a formation of the hybrid material HPS.

In Figure 4b, as compared with that of the unmodified SiO_2_, it is obvious that, after VTMO modification, the width peak of the SiO-H stretching band at 3489 cm^−1^, the anti-symmetric stretching vibration of SiO-H, and the water’s H-O-H bending vibration near at 1635 cm^−1^ on the surface of silica nanoparticles decrease significantly. These two peaks basically disappeared in the modified SiO_2_, indicating that Si-OH has fully condensed into Si-O-Si bonds during the process of hydrophobic modification [35,37]. Compared with that of the pure SiO_2_, small peaks at 2964 cm^−1^ emerged in the FTIR spectrum of the VTMO-SiO_2_, which corresponds to characteristic Si-C stretching vibrations [38]. Such findings further highlight that the hydroxyl groups have been replaced successfully by the hydrocarbyl groups, due to the introduction of VTMO.

### 3.3. Thermogravimetric Analysis

**Figure 5:**
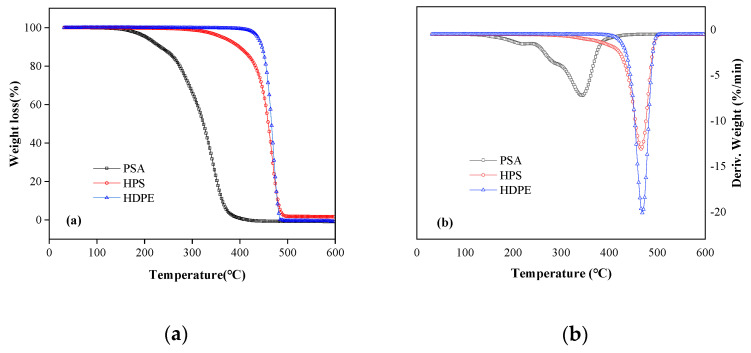
Thermal analysis of different materials: (**a**) thermogravimetry (TG) and (**b**) derivative thermogravimetry (DTG).

The TGA and DTG results of the copolymer PSA, pure HDPE and the HDPE composite with addition of PSA and SiO_2_ are shown in Figure 5. For the copolymer PSA, as measured in untreated copolymer, there is 6.5% weight loss at the temperature range of 130–210 °C, due to the removal of unreacted monomers residuals. The main degradation stage of copolymer is in the range of 240–410 °C, and its maximum pyrolysis rate occurs at 341°C, with weight loss reaching 87.4%. The PSA is completely degraded at 420 °C, shown in Figure 5a. According to the DTG of PSA (Figure 5b), the first decomposition shoulder peak at 215 °C is the pyrolytic polymerization of the polymer, and the second major decomposition peak at 341 °C is the polymer decomposition.

The DTG curve shows strong and sharp endothermic peak at 468 °C for the pure HDPE and 466 °C for the composite (Figure 5b). This fact implies that the thermal decomposition temperature of the prepared composite is not affected by the addition of acrylate with lower decomposition temperature, which demonstrates excellent thermal stability. In addition, there is only one obvious decomposition peak in the DTG curve of the HPS composite (Figure 5b), indicating that the polymer may cross-link under the action of residual siloxane and initiator in the process of melt blending [39]. Cross-linking of polymers is expected to minimize solvent uptake, avoid crystallinity and fix their surface in a solution environment [40].

### 3.4. SEM Analysis

**Figure 6:**
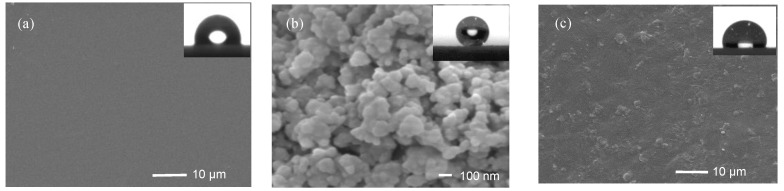
SEM images of different samples: (**a**) PSA10; (**b**) VTMO-SiO_2_; and (**c**) HPS5.

The typical SEM images of different samples are shown in Figure 6. It can be seen that the surface of PSA10 is smooth, without obvious surface features (Figure 6a). The particle size of hydrophobically modified nano silica powder is about 100 nm (Figure 6b) and the static water CA is tested up to 154.5°, which can be considered as superhydrophobic surface. There is a slight aggregation of particles on the surface of HDPE composite after adding polymer PSA10 and superhydrophobic nano-SiO_2_ (Figure 6c), which may be attributed to the Van der Waals bond between nanoparticles [27]. The agglomerated nanoparticles are distributed in bright spots in the dark HDPE matrix, and the distribution of particles in the whole matrix is relatively uniform, which may lead to an increase in cavitation in the material surface, resulting in an increase of CA to further demonstrate an excellent hydrophobicity [33].

### 3.5. Acid and Alkali Resistance

**Figure 7:**
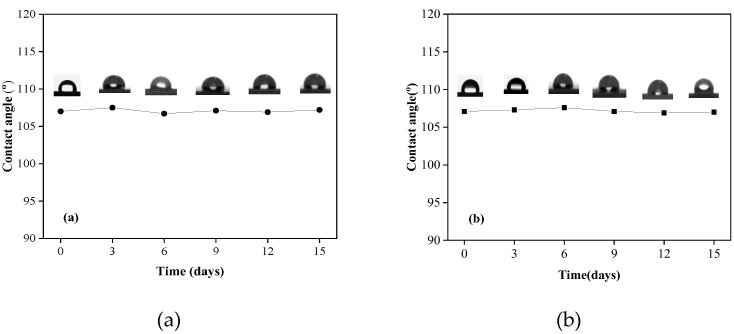
Contact angle changes after immersed in simulated leachate (**a**) pH = 2.0, (**b**) pH = 12.0.

It is significant to test the acid and alkaline resistance of the prepared material in order for possible engineering practice on waste water piping. High concentrated acid solution (HCl, pH = 2) and basic solution (NaOH, pH = 12) have been introduced to assess the stability of the hydrophobic ability of the HPS5 composite under extreme environment. After being immersed in the acid and alkali solutions for 15 days, there is no obvious changes of CA, shown in Figure 7. This indicates that the prepared composite material shows certain potentials in acid and alkaline resistance.

### 3.6. Anti-Scaling Properties

A comparative test is designed to investigate the adhesion of the material surface to the fouling by application of scale dust washing. The dried scale samples were taken from the landfill leachate pipeline in Chengdu city, ground and passed through 100 mesh sieve, then sprayed on the surface of the unmodified HDPE resin and the prepared composite, respectively. After the water droplets flowed through the layer of the scale samples, the residuals on the material surface were investigated.

**Figure 8:**
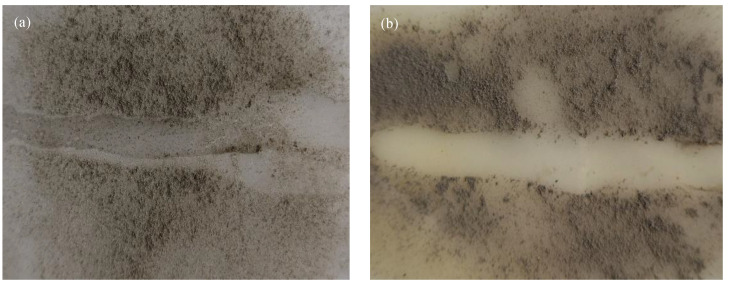
Anti-scaling properties of (**a**) HDPE and (**b**) HPS5 composite.

Figure 8 shows that nearly all the scaling dusts on the surface of the prepared composite are washed away by water droplets, whilst there are still certain scaling dusts remained on the surface of HDPE. It is indicated that the HDPE substrates are prone to scaling. Existing studies have addressed that low surface energy substrate is effective to decrease fouling adhesion [25,41,42]. This study identifies that the surface free energy of the prepared composite is lower than that of the commercial HDPE pipe (shown in Table 4), indicating better scaling resistance.

**Figure 9:**
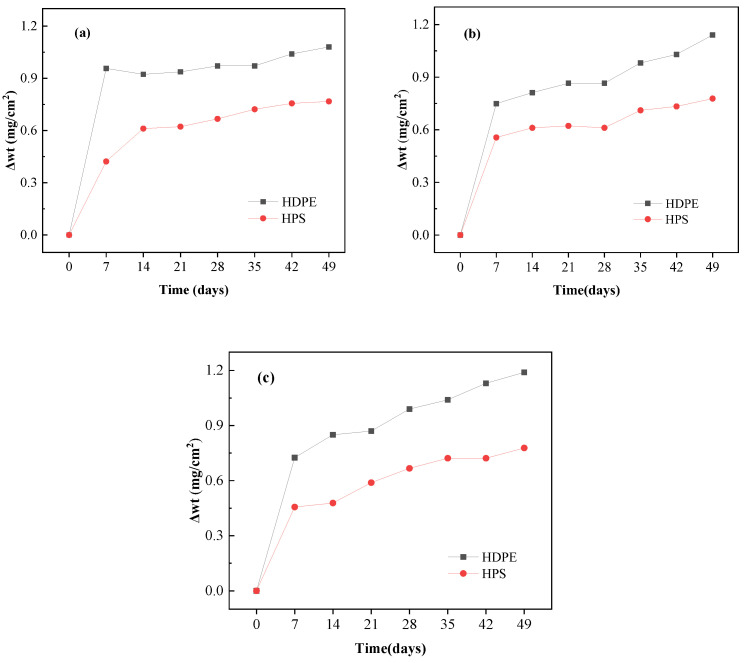
Weight of material samples in simulated leachate under different conditions: (**a**) pH = 4.0; (**b**) pH = 6.8; (**c**) pH = 10.0.

The scales are measured by comparing the weight of HDPE samples and the composite samples, as shown in Figure 9. Both of the two materials occurs weight increase after soaking in the same simulated leachate, and the variation of HDPE is greater than that of the composite material. Among them, the HDPE shows significant weight increase in the first period when placing into the simulated leachate in pH of 4 (see Figure 9a). The reason may be the fact that the inorganic filler in HDPE is corroded under strong acid environment [43].

**Figure 10:**
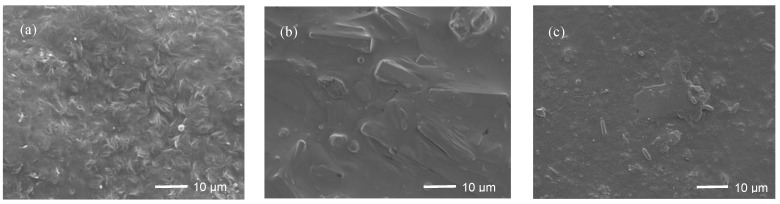
SEM images of the material samples immersed in the simulated leachate solution (pH = 4.0) for 14 days: (**a**) Original HDPE; (**b**) HDPE after immersion; (**c**) HPS5 composite.

**Figure 11:**
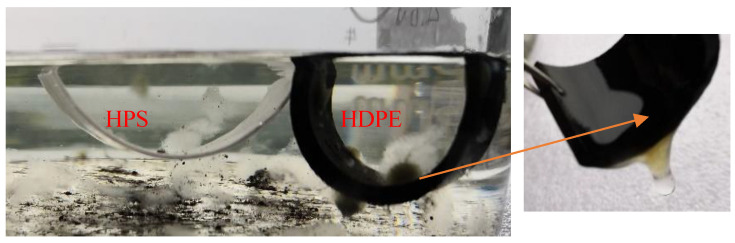
Material samples immersed in the simulated leachate (pH = 4.0).

Figure 10 shows the SEM images of different materials immersed in the simulated leachate with pH of 4.0 after 14 days. There are columnar or square crystals on the surface of HDPE sample (Figure 10b). Such crystals may be encapsulated by the bio-films, since it has been further identified by the surface of commercial HDPE pipe which is adhered to gelatinous substances, shown in Figure 11. A possible reason for this may be that the immersion experiment is not implemented under an aseptic environment, by which the organic constituents in the simulated leachate, e.g., glucose, may provide carbon source and phosphorus as the nutrients to nourish microorganisms. They are further adhered to the immersed material samples to promote their metabolisms, ultimately forming bio-films. However, there is no obvious crystal formation on the surface of the prepared composite, only a few rod-shaped and lamellar scales (Figure 10c). This may be related to the good hydrophobicity and low surface free energy of the prepared composite. The hydrophobic material surface may inhibit crystal nucleation, and thus has excellent anti-scaling properties [40,44,45].

### 3.7. Discussion

In this work, copolymers have been polymerized by bulk polymerization without addition of organic solvents, which may decrease environmental impact during the preparation process. The addition of modified nano-silica is identified to improve the wear resistance, hydrophobicity and thermal stability of the prepared composite. The bifunctional groups of siloxanes react with nano-silica and acrylate, to produce a chemical bond between polymer and filler. Thus, the distribution of filler in the polymer is more uniform and stable [46].

The surface energy of the composite is as low as 23.5 mN/m when the content of PSA and nano-SiO_2_ is added as 15%, 5%, respectively. Azimi et al. identified that if surface energy is below 25 mN/m, the nucleation of scale will be significantly decreased [11]. Such conclusion may imply that our prepared composite has potentials in resistance of scaling deposition. Furthermore, the study fabricates the organic/inorganic cross-linked composite materials to replace conventional anti-scaling coatings, which may improve the lifespan of pipe materials.

The immersion experiment results shows that the scaling on the surface of the prepared composite is less than that of commercial HDPE pipe. It further verifies that the anti-scaling performance may have strong relations with the hydrophobicity and surface energy regarding the prepared composite, i.e., the better hydrophobicity and the lower surface energy may result in less scaling happened.

The composite demonstrates a strong adaptability to the extreme acid and alkaline environment, indicating it may have wide applications to the collection and transport of wastewater from various emissions sources. However, the mechanism of scale formation is still uncertain, which lays out a room for investigation on interaction between scaling and material surface, especially on how leachate affects such interaction.

## 4. Conclusions

This study prepares a low surface energy silicified acrylate copolymer by bulk polymerization. Such copolymer is added by melt blending with HDPE resin and different content of hydrophobic nano-SiO_2_ to prepare a nano-composite, entitled HDPE/PSA/SiO_2_ composite. Through the characterization of FTIR, TG, CA, SEM, etc., it is confirmed that PSA and hydrophobic nano-SiO_2_ are successfully synthesized, such that there is no other organic solvent in addition to the reaction monomer. The thermal analysis results show that the addition of silicified acrylate polymer and nano-SiO_2_ have little influence on the thermal decomposition temperature, to confirm that the prepared composite has good thermal stability. The surface free energy is decreased to 23.5 mN/m for the composite, and its water contact increases to 107.1°. It thus implies that 5% hydrophobic nanoparticles is the best percentage of addition.

HDPE/PSA/SiO_2_(5%) is selected to compare its acid and alkali resistance with the common commercial HDPE pipe material. The composite has good wear resistance and strong adaptability to extreme acid and alkali environment. Through an immersion experiment by placing the prepared composite and the commercial HDPE samples into the simulation leachate, the results show that the former has better anti-scaling property due to its low surface energy and wettability. It is thus indicated that the composite material may have perspectives on engineering application to various wastewater piping transport.

There is room for further study. First, the interaction between scaling particles and material surface, as well as how various water quality may affect such interaction still need to be investigated. Further study should center on the analysis of possible mechanism of scaling. In addition, other properties of composite materials, such as mechanical properties, aging resistance, etc., are expected to be tested to improve its feasibility in engineering application. Furthermore, it is also important to consider the verification of the practical application value of the material in the simulated wastewater flow pipeline experiment.

## Figures and Tables

**Table 1:** Compositions of polymers of silicified acrylate copolymer with different monomers content.

PSAs	Proportion of Constituents (%)		
	VTMO	BMA	GMA
PSA0	0	70	30
PSA1	1	70	29
PSA2	2	70	28
PSA5	5	70	25
PSA10	10	70	20
PSA15	15	70	15
PSA20	20	70	10

**Table 2:** Different mixture ratios for the composite preparation.

Samples Composition	Labeling	Proportion of Constituents (%)		
		PSA	HDPE	VTMO-SiO_2_
HDPE/PSA/SiO_2_(1%)	HPS1	9	90	1
HDPE/PSA/SiO_2_(5%)	HPS5	15	80	5
HDPE/PSA/SiO_2_(9%)	HPS9	11	80	9

**Table 3:** Water quality parameters for the simulated leachate.

	Main Parameters				
	COD (mg/L)	pH	Ca^2+^ (mg/L)	Suspended Solids (mg/L)	References
Common leachate	5800–117,000	3.8–9.0	250–7200	20–280	[1,9,32]
Simulated leachate	35,000	4.0–10.0	2500	100	

**Table 4:** Contact angle of different PSAs copolymers.

PSAs	CA (°)
PSA0	92.1 ± 1.2
PSA1	97.5 ± 1.0
PSA2	98.8 ± 0.8
PSA5	100.5 ± 1.0
PSA10	104.9 ± 1.5
PSA15	101.9 ± 1.0
PSA20	102.7 ± 0.9

**Table 5:** Contact angle and surface free energy of different substrates.

Substrates	CA (°)		$\sigma_{solid}(\mathrm{mN}/m)$
	**H_2_O**	**CH_2_I_2_**	
HDPE	86.6	45.4	37.0
HPS1	106.1	56.5	32.2
HPS5	107.1	69.7	23.5
HPS9	103.5	53.3	33.8
PSA10	104.9	54.7	33.0
